# In Vitro Antimicrobial Activity of the Novel Antimicrobial Peptide OMN51 Against Multi-Drug-Resistant *Pseudomonas aeruginosa* Isolated from People with Cystic Fibrosis †

**DOI:** 10.3390/jcm14155208

**Published:** 2025-07-23

**Authors:** Moshe Heching, Moshe Cohen-Kutner, Haim Ben-Zvi, Liora Slomianksy, Elital Chass Maurice, Noa Nur Maymon, Shira Mandel, Michal Oholy, Rony Moses, Michal Lavon, Katherine Kaufman, Orel Mayost Lev-Ari, Tamar Shachar, Joel Weinberg, Mordechai R. Kramer, Niv Bachnoff

**Affiliations:** 1 Pulmonology Institute and Adult CF Center, Rabin Medical Center, Beilinson Hospital, 39 Jabotinsky Street, Petach Tikva 4941492, Israel; 2 School of Medicine, Faculty of Medical and Health Sciences, Tel Aviv University, Tel Aviv 6997801, Israel; 3 Omnix Medical Ltd., High-Tech Village, Givat-Ram Campus, Jerusalem 9190401, Israel; 4 Clinical Microbiology Laboratory, Rabin Medical Center, Beilinson Hospital, Petach Tikva 4941492, Israel

**Keywords:** *Pseudomonas aeruginosa*, multi-drug-resistant, antibiotics, antimicrobial peptide, in vitro, bactericidal, OMN51, cystic fibrosis, infections

## Abstract

**Background:** People with cystic fibrosis (pwCF) frequently suffer from chronic lung infections, with *Pseudomonas aeruginosa* being the predominant pathogen contributing to disease progression and morbidity. The increasing prevalence of multi-drug-resistant (MDR) *P. aeruginosa* has diminished treatment options. Antimicrobial peptides (AMPs) have emerged as promising alternatives to conventional antibiotics due to their unique membrane-targeting mechanisms. OMN51, a novel bioengineered AMP derived from capitellacin, was evaluated for antimicrobial activity against *P. aeruginosa* in sputum samples from pwCF. This study aimed to compare the bactericidal effects of OMN51 with those of a range of conventional antibiotics known to have activity against *P. aeruginosa* clinical isolates derived from pwCF. **Methods:** *P. aeruginosa* clinical isolates were obtained from fifty-six unique sputum cultures of pwCF at a tertiary-university-affiliated hospital. Minimum inhibitory concentrations (MICs) of OMN51 and comparator antibiotics were determined using broth microdilution. Antimicrobial susceptibility was evaluated using the Kirby–Bauer disc diffusion method. **Results:** OMN51 demonstrated in vitro bactericidal activity across all *P. aeruginosa* isolates, including MDR strains. MIC values for OMN51 ranged from 4 to 16 µg/mL, with no observed resistance or cross-resistance. Comparative analysis revealed the superior efficacy of OMN51 compared with conventional antibiotics. **Conclusions:** OMN51 exhibits robust in vitro activity against MDR *P. aeruginosa*, supporting its candidacy as a therapeutic agent for MDR *P. aeruginosa-* associated infections. Further studies are warranted to assess pharmacokinetics and in vivo safety and efficacy. OMN51 represents a first-in-class, membrane-targeting therapeutic showing promise against MDR *P. aeruginosa*.

## 1. Introduction

Cystic fibrosis (CF) is a chronic genetic disease marked by persistent respiratory infections and progressive decline in lung function. CF is caused by a mutation in the CFTR gene, which regulates chloride ion concentrations across epithelial membranes. Dysfunction in ion regulation leads to the accumulation of thick mucus in the airways, which are a nidus for respiratory pathogens, which causes recurrent infections, inflammation, and harmful airway remodeling. Early detection and treatment through airway clearance and antibiotics are critical to prevent diffuse bronchiectasis and progressive decline in lung function. Advances in medical care for people with cystic fibrosis (pwCF) have improved both overall healthspan and lifespan. However, respiratory failure due to infected bronchiectasis remains the leading cause of death in pwCF [1].

Current therapeutic approaches for CF combine CFTR modulator therapy in eligible pwCF with supportive management targeting pulmonary infections, principally airway clearance, anti-inflammatory agents, and antibiotics. Antibiotics in various delivery formats are the mainstay treatment to delay lung function deterioration in pwCF. Widespread overuse of antibiotics in CF bronchiectasis has led to ever-increasing resistance patterns of microbes to existing antibiotics [2]. In pwCF, risk of mortality is independently linked to the presence of multi-drug-resistant (MDR) bacteria, which increase exacerbations, reduce antibiotic effectiveness, and lead to progressive lung dysfunction [3]. MDR is defined as non-susceptibility to at least one agent from three or more antimicrobial classes [4]. In 2021, the global mortality burden from MDR bacteria was estimated to exceed one million deaths annually, with a global morbidity burden exceeding forty million disability-adjusted life years [5].

A substantial therapeutic gap persists in developing antibiotics specifically effective against MDR Gram-negative bacteria. Four of the six ESKAPE pathogens, identified as priority pathogens by the World Health Organization due to their global health threat, are Gram-negative. These pathogens include *Pseudomonas aeruginosa*, an opportunistic pathogen associated with progressive bronchiectasis, which has historically been more difficult to eradicate. Despite advances in antimicrobial therapy, treatment options for *P. aeruginosa* remain limited, particularly due to its adaptability and acquired resistance mechanisms [6,7].

*P. aeruginosa* has various virulence factors that can adapt to its host and overcome immune system barriers, precipitating chronic lung infections [8]. Virulence factors are molecules produced by bacteria that allow them to evade or suppress the host immune response, thereby increasing bacterial pathogenicity. As such, treating *P. aeruginosa* is a formidable challenge due to the impermeability of its outer membrane, combined with its virulence factors: efflux pumps, enzymatic degradation of antibiotics, and biofilm formation [9]. Enzymatic degradation of antibiotics is a primary mechanism by which bacteria achieve resistance to antimicrobial agents, releasing enzymes that chemically modify or hydrolyze antibiotics, rendering them inactive [10]. This is further complicated by the propensity of *P. aeruginosa* to form biofilms in the CF lung, creating an extracellular matrix that shields bacteria from both immune responses and antibiotics, promoting chronic infection and resistance development [7].

*P. aeruginosa* is the most prevalent and virulent microbial pathogen in pwCF, contributing to pulmonary exacerbations and increased morbidity [8,11]. *P. aeruginosa* is the principal pathogen responsible for chronic lower airway infection and progressive lung function decline in pwCF, and signals a transition to a more severe disease phenotype with increased morbidity and mortality [12]. *P. aeruginosa* infection exacerbates CF lung disease through a maladaptive immune response causing chronic inflammation, the recruitment of neutrophils, and the production of virulence factors and proteases, which are immunogenic and damage the lung tissue [8]. The emergence of MDR *P. aeruginosa* strains has complicated treatment strategies. In the presence of MDR bacteria, conventional antibiotic options are limited and innate host immune responses cannot effectively eradicate the bacteria. The vicious cycle of infection and inflammation accelerates lung function decline characteristic of bronchiectasis, underscoring the need for therapeutics that can simultaneously target bacterial clearance and modulate inflammation [13,14]. New therapeutic options are urgently needed to address this growing concern.

Antimicrobial peptides (AMPs) are innate defense mechanisms produced by all living organisms, acting as the first line of defense against pathogenic infections [15]. AMPs represent promising alternatives to conventional antibiotics due to their ability to disrupt bacterial membranes, reducing the likelihood of resistance development [16]. However, the adoption of AMPs for antibiotic applications has been limited because many AMPs can be easily hydrolyzed and degraded by natural cellular proteases [17]. To overcome these limitations, AMPs need to be bioengineered for enhanced stability and efficacy to effectively target microbes [18,19].

Beyond their direct antimicrobial effects, AMPs exhibit diverse immunomodulatory effects, including the ability to recruit immune cells and modulate inflammatory cytokine production [20]. AMPs’ pleiotropic effects in immunomodulation may enhance the immune system, mitigating the chronic inflammation that causes lung damage [20]. Moreover, advances in AMP engineering have enabled the development of AMPs with enhanced resistance to enzymatic degradation, improved selectivity for bacterial cells, and reduced toxicity to eukaryotic host cells, paving the way for clinical translation [21].

Capitellacin, a beta-hairpin 20-amino acid AMP secreted by the marine polychaeta (segmented worms) *Capitella teleta*, is a naturally occurring antimicrobial peptide. Capitellacin has been demonstrated in vitro to have broad-spectrum antimicrobial activity against Gram-negative bacteria, including MDR *P. aeruginosa* [22,23]. While harmful to bacteria, capitellacin has low cytotoxicity to mammalian cells in its native form [22,23]. OMN51 is a novel bioengineered synthetic AMP developed by Omnix Medical Ltd., designed as a modified analog of capitellacin. We explored the in vitro effectiveness of OMN51 in MDR *P. aeruginosa*. This study aimed to compare the bactericidal effects of OMN51 with those of a range of conventional antibiotics known to be effective against *P. aeruginosa.*

## 2. Materials and Methods

Bacterial isolation: *P. aeruginosa* clinical isolates were collected from anonymized pwCF sputum cultures at the Rabin Medical Center (RMC) adult CF clinic, a tertiary university-affiliated hospital, between August 2023 and August 2024. From these cultures, unique bacterial isolates were recovered, inoculated, and cultured on blood agar, chocolate agar, MacConkey agar, and Burkholderia Cepacia Selective Agar plates (HyLabs, Rehovot, Israel). Plates were incubated under temperature and atmospheric conditions considered optimal for the growth of *P. aeruginosa* (35–37 °C, 1 ATM). Isolates were identified based on their morphology using standard microbiological techniques, including positive oxidase reaction, assessment of pigment production, ability to grow at 42 °C, and non-fermentative metabolism on triple sugar iron agar, followed by final identification with Bruker Biotyper Matrix-Assisted Laser Desorption/Ionization Time-of-Flight Mass Spectrometry (MALDI-TOF MS) system (Bruker Daltonics, Billerica, MA, USA). All isolates were tested by broth microdilution using non-cation-adjusted Mueller–Hinton broth to determine the antimicrobial susceptibility and resistance profile using the Clinical and Laboratory Standards Institute (CLSI) guidelines.

Peptide synthesis: Capitellacin is a beta-hairpin AMP derived from the marine polychaeta *Capitella teleta* (amino acid sequence: SPRVCIRVCRNGVCYRRCWG) [23]. OMN51 is a bioengineered derivative of capitellacin. OMN51 is a C-terminal amidated 20-amino acid long cyclic peptide, proprietarily modified from the native capitellacin amino acid sequence, with a molecular weight of 2320 Da. OMN51 is bioengineered to reduce the two disulfide bonds found naturally in capitellacin to a single disulfide bond by replacing two cysteine residues with alanine, resulting in the loss of one disulfide bond. As a result of this structural change, OMN51 has enhanced antimicrobial activity while maintaining its stability against proteolytic degradation, yet retains the selectivity and known safety profile of its analog capitellacin. OMN51 is synthetized as an acetate salt, using only natural L-amino acids. OMN51 is produced in several stages: solid phase synthesis process and peptide resin cleavage process, followed by cyclization, purification, salt exchange, and lyophilization processes. The single disulfide bond enables a more streamlined and cost-effective synthesis process [24].

**Figure 1:**
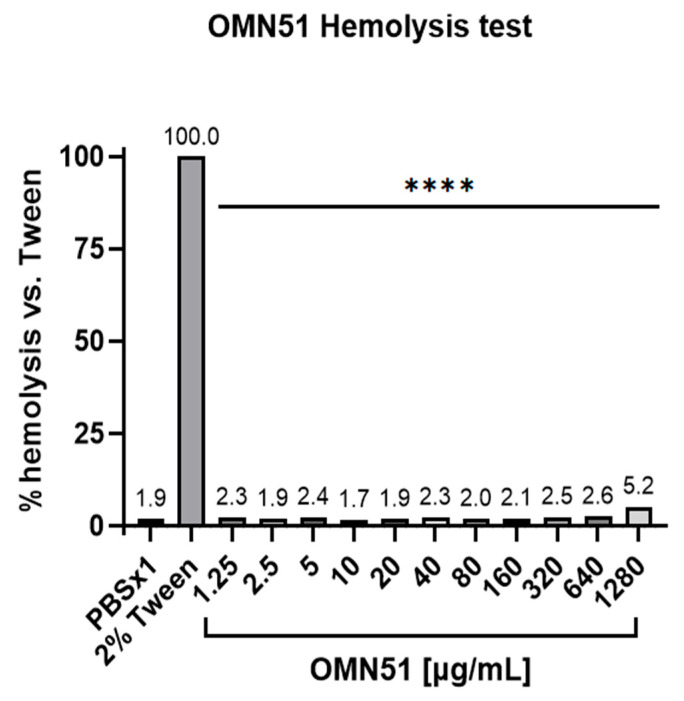
Erythrocyte hemolysis assay. Suspensions of 10% mouse RBCs were exposed to increasing concentrations of OMN51. The positive control group was treated with 2% Tween 20, with the results deemed a reference for 100% hemolysis. After a 1 h incubation, the relative amount of free hemoglobin was assessed as an indication of erythrocyte hemolysis, compared with the free hemoglobin values obtained with the positive control, and presented as a percentage of hemolysis vs. 2% Tween (**** *p* < 0.0001 for all OMN51 concentrations).

Cytotoxicity assay: OMN51 was assessed for cytotoxicity to mammalian cells using an erythrocyte hemolysis assay. Red blood cell (RBC) suspension was prepared from whole blood extracted from the heart of Hsd:ICR (CD-1) mice (Envigo, Huntingdon, Cambridgeshire, UK). This study was carried out in accordance with relevant guidelines and regulations, and was approved by the Hebrew University Ethics Committee for the Care and Use of Laboratory Animals. Blood was collected in a 24 U/mL heparin tube to prevent coagulation. RBCs were then washed with Phosphate Buffered Saline (PBS; Biological Industries, Sartorius AG, Göttingen, Germany) and centrifuged at 200× *g* for 10 min at 20 °C. This operation was repeated three times, and the remaining RBCs were resuspended in saline or PBS to form a 10% RBC suspension. Suspensions of 10% RBCs were exposed to increasing concentrations of OMN51 and compared with 2% Tween 20 as a positive control and PBS as a negative control. After 1 h incubation at 37 °C while agitating at 100 rpm, experiment tubes were centrifuged for 10 min at 200× *g* at room temperature. An amount of 100 μ/L of supernatant was extracted from each tube and tested according to the Hemoglobin Assay Kit (Sigma-Aldrich, Merck KGaA, Darmstadt, Germany). The relative amount of free hemoglobin (Hgb, mg/dL), as an indicator of erythrocyte hemolysis, was assessed spectrophotometrically by measuring the absorbance of released hemoglobin at an optical density of 400 nm (Figure 1) [24].

Proteolytic analysis: The susceptibility of OMN51 to proteolytic degradation was assessed in a proteolytic study, demonstrating the stability of OMN51 and its resistance to proteolytic degradation [24].

MIC testing: Bacterial inoculum was prepared from isolated colonies, dispensed in culture wells, and treated with OMN51. OMN51 solutions were prepared at fivefold the intended final concentrations, corresponding to final concentrations in the minimum inhibitory concentration (MIC) plate wells of 0.5–128 µg/mL OMN51 in Mueller–Hinton broth. The final concentration was achieved after manually pipetting the concentrated OMN51 solution into the culture wells. Tobramycin and ceftazidime were used as a control for verifying bacterial viability. Antibiotic solutions were prepared similarly at fivefold the intended final concentrations, achieving final concentrations in the MIC plate wells of 0.03–8 µg/mL for tobramycin and 0.06–16 µg/mL for ceftazidime. As per the CLSI guidelines for MIC testing, the bacterial inoculum concentration was standardized to approximately 5 × 10^5^ colony-forming units (CFU)/mL (range, 2–8 × 10^5^ CFU/mL) in each well of the microdilution plate. This concentration was achieved by preparing a bacterial suspension equivalent to 0.5 McFarland standard (approximately 1–2 × 10^8^ CFU/mL), followed by a 1:300 dilution into the test medium.

Test plates were incubated according to CLSI guidelines and assessed visually. When visual reading was not feasible, plates were read by a microplate reader (Tecan Group Ltd., Männedorf, Switzerland) at an optical density of 625 nm. The bacteria were subjected to MIC tests using the broth microdilution method following the principles outlined in CLSI guidelines. MIC values were determined at the lowest concentration that completely inhibits growth of the inoculated bacteria as detected by either the unaided eye or the Tecan microplate reader. MIC results were interpreted according to current CLSI guidelines and compared with the antimicrobial activity of other antibiotics on the *P. aeruginosa* bacterial strains. OMN51 MIC values were reported as MIC_50_ and MIC_90_, representing the lowest concentrations required to inhibit the growth of 50% and 90% of the tested isolates, respectively.

Susceptibility testing: Susceptibilities for antimicrobials were determined by the modified Kirby–Bauer disc diffusion methodology following criteria used by the RMC laboratory based on CLSI standardized protocols for antimicrobial sensitivity testing. Results were compared against reference strains with known susceptibility profiles to ensure the accuracy of the testing process. Results were interpreted using breakpoints defined by CLSI, which categorize bacteria as susceptible, intermediate, or resistant based on the MIC. The choice of antimicrobials for testing was guided by clinical relevance and local resistance patterns and antibiograms. 

Bactericidal assessment: Time-kill kinetics assays were performed to evaluate bactericidal activity at various OMN51 concentrations on both tobramycin-sensitive and tobramycin-resistant *P. aeruginosa* isolates.

This study was approved by the RMC institutional review board.

## 3. Results

We tested the efficacy of the novel AMP OMN51 in vitro on isolates obtained from pwCF to assess the bactericidal activity of OMN51 against *P. aeruginosa* clinical isolates with diverse resistance profiles. A total of fifty-six (56) *P. aeruginosa* isolates were collected and tested (twelve additional isolates were unculturable on agar plates and excluded from the analysis). OMN51 was active against 100% of *P. aeruginosa* isolates with a narrow MIC range (4–16 µg/mL) as compared with the other antimicrobial agents tested. OMN51 was effective against susceptible, resistant, and MDR *P. aeruginosa* clinical isolates, with a MIC range of 4–16 µg/mL for MDR isolates, as reported in Table 1.

OMN51 resulted in a potent inhibitory effect on *P. aeruginosa* bacteria with a MIC of ≤16 µg/mL across the tested strains, irrespective of their resistance profile. OMN51 displayed an effective response rate against *P. aeruginosa* isolates tested in this study with a MIC range of 4 to 16 μg/mL, a MIC_50_ of 8 μg/mL, and a MIC_90_ of 16 μg/mL. The percentage of resistant *P. aeruginosa* isolates for the antimicrobial comparators is reported in Table 2.

Time-kill kinetics demonstrated that OMN51 exhibited rapid bactericidal activity against both tobramycin-sensitive and tobramycin-resistant *P. aeruginosa* isolates, achieving complete eradication within 1 h at twofold the MIC for tobramycin-sensitive isolates and within 8 h at fourfold the MIC for tobramycin-resistant isolates. OMN51 was assessed for cytotoxicity to mammalian cells using an erythrocyte hemolysis assay, and the results demonstrated minimal level of hemolytic activity at all concentrations tested (Figure 1).

## 4. Discussion

Our results provide a proof of concept for the bactericidal effects of OMN51 in vitro against clinical isolates of *P. aeruginosa*. OMN51 consistently exhibited a MIC range of 4–16 µg/mL, with effectiveness across all tested clinical isolates. OMN51 demonstrated the same level of efficacy against both sensitive and MDR *P. aeruginosa* isolates. The peptide’s activity was unaffected by resistance mechanisms that compromised the efficacy of other antimicrobials, indicating that no resistance or cross-resistance to the peptide was observed in the *P. aeruginosa* clinical isolates. These observations confirm the low propensity for resistance development against OMN51.

OMN51 is a beta-hairpin antimicrobial peptide derived from capitellacin, an AMP of the innate immune system of *Capitella teleta*. Although the mechanism of action of capitellacin has not been fully elucidated, beta-hairpin AMPs, such as capitellacin, primarily target the cellular membrane [22]. Rather than binding to a specific molecular target on the cell, membrane-active AMPs exhibit a detergent-like mechanism of action, disrupting the bilayer lipid membrane [23]. In contrast with conventional antibiotics, which depend on a specific biochemical site of action or interaction, AMPs like OMN51 cause physical damage to the bacterial membrane structure [25]. When a sufficient number of AMPs bind to the membrane, they induce strain, leading to membrane disruption. The membrane disruptive mechanism of AMPs has been demonstrated in various studies to be concentration dependent, where a threshold density of bound peptides culminates in membrane perturbations and destabilization [26]. This compromises the integrity of the bacterial outer membranes, disrupts cellular electrochemical gradients, depletes the proton gradient, causes cell swelling, increases intracellular osmotic pressure, and results in bacterial cell lysis and rapid bacterial death [27].

The broad membrane-disruptive action of AMPs targets not only the bacteria themselves but potentially also bacterial biofilms. Biofilms, which consist of an extracellular matrix of polysaccharides surrounding bacteria, present a major barrier to antibiotic penetration and immune clearance in *P. aeruginosa*-infected airways. AMPs have shown effectiveness in penetrating biofilm matrixes and enhancing antibiotic susceptibility in vitro, suggesting an important role for AMPs in addressing chronic *P. aeruginosa* infections where biofilms predominate [28].

Through this unique mechanism, AMPs can distinguish between host cells and bacteria based on the lipid composition and net electric charge of the bacterial membrane, making them inert against non-bacterial host cells [25]. Due to their specificity for the cellular membrane, synthetic AMPs can be designed with low cytotoxicity towards mammalian cells [29]. Of note, OMN6, a bioengineered cyclic 40-amino acid AMP derived from Cecropin A and developed by Omnix Medical Ltd., has demonstrated potent antimicrobial activity against MDR Gram-negative bacteria, including *Acinetobacter baumannii* [30]. OMN6 exerts a bactericidal effect by selectively disrupting bacterial membranes, significantly lowering the risk of resistance development [31].

The membrane-disruptive mechanism of AMPs provides a unique advantage over conventional antibiotics, which depend on specific biochemical targets. Unlike beta-lactams and fluoroquinolones, which are prone to resistance due to enzymatic degradation or efflux pump activity, antimicrobial peptides like OMN51 cause rapid cell lysis by targeting the bacterial membrane [32]. This mechanism reduces the likelihood of resistance development, as bacteria would need to undergo significant structural changes to evade its effects. Since AMPs target evolutionarily conserved components of the bacterial membrane, resistance development would require extensive structural reconfiguration of the membrane, demanding multiple mutations over an extended period [33]. This is particularly significant given the increasing prevalence of carbapenem- and colistin-resistant *P. aeruginosa*, which dramatically limit available treatment options [34].

Notably, when tested against conventional antibiotics, OMN51 in vitro antimicrobial activity remained unaffected by various pre-existing resistance mechanisms, including carbapenem resistance. Our results showed no evidence of cross-resistance between OMN51 and commonly used antibiotics, further supporting its potential as a novel therapeutic option. The ability of OMN51 to retain activity regardless of resistance profiles indicates that, similar to other AMPs [35], it is not susceptible to the primary resistance mechanisms that affect conventional antibiotics. Furthermore, OMN51 demonstrated activity against both susceptible and multi-drug-resistant strains, highlighting its broad-spectrum efficacy.

The time-kill assays reinforced these findings, demonstrating that, for the tobramycin-sensitive isolate, OMN51 at twofold the MIC achieved total bacterial clearance within 1 h, and for the tobramycin-resistant isolate, OMN51 at fourfold the MIC achieved total bacterial clearance within 8 h. This rapid bactericidal effect is crucial in managing acute exacerbations in pwCF, where prompt bacterial clearance can prevent irreversible lung damage.

AMPs offer a unique advantage in that a single molecule can exert its effects through multiple distinct mechanisms of action. While the most common mechanism involves direct disruption of microbial cell membranes, other mechanisms include intracellular targeting, non-lytic membrane activity, and anti-biofilm effects. This multimodal activity not only enhances antimicrobial potency but also reduces the likelihood of resistance development. Bacteria are less likely to simultaneously acquire the multiple mutations required to overcome such diverse mechanisms.

Co-administration of AMPs with traditional antibiotics may help overcome or prevent antibiotic resistance, potentially reducing the required dosages and associated toxicity. Recent studies have explored synergies between AMPs and conventional antibiotics, enhancing the efficacy of combined antibiotic therapies [36]. This is particularly relevant in CF therapy, where combined treatment with synergistic antibiotics against *P. aeruginosa* is well established [7].

Beyond synergy with antibiotics, AMPs may enhance immune responses by interacting synergistically with host immune components. AMPs may have immunomodulatory effects that enhance host defenses, including macrophage activation and cytokine modulation, potentially contributing to clinical benefit in chronic pulmonary infections [20]. AMPs have been shown to modulate immune responses and reduce inflammation, both of which are key factors in CF lung disease progression. The persistent cycle of infection and inflammation in CF airways contributes to lung deterioration, and a treatment capable of both direct bacterial killing and immune modulation would be particularly beneficial [37]. While further studies are needed to assess these immunomodulatory effects of OMN51, prior research on AMPs suggests that OMN51 could offer additional benefits beyond pathogen clearance [20].

Further studies are planned to assess the optimal delivery mechanism for OMN51. Specifically, OMN51 has the potential to be administered in inhaled formulation, similar to tobramycin and aztreonam, for both acute and maintenance treatment in hospital and home settings. Chronic inhaled antibiotic administration is the standard of care for managing chronic *P. aeruginosa* infection in pwCF. This is supported by significant evidence for inhaled antibiotics causing a decrease in the *P. aeruginosa* density in the sputum, as well as improvements in respiratory symptoms, quality of life, and lung function [38]. The inhaled delivery method is well suited for pulmonary infections, allowing the active ingredient to reach high therapeutic concentrations in the lung, with low systemic exposure that minimizes adverse effects.

This study has various limitations, as it is limited to in vitro assays, and further research is needed to determine the pharmacokinetic and pharmacodynamic properties of OMN51. Further, while our results provide preliminary evidence of OMN51 in vitro efficacy, future planned studies will evaluate its in vivo safety, toxicity, efficacy, and potential for systemic and inhaled administration.

Additional studies investigating OMN51’s efficacy in animal models of chronic *P. aeruginosa* infection are critical to validate these findings in vivo and to explore pharmacokinetics, tissue distribution, and dosing regimens. Moreover, assessing OMN51’s effects on biofilm disruption and host immune modulation in vivo will provide valuable insights for clinical translation. Toxicology and safety studies will be essential to ensure therapeutic windows that maximize antimicrobial activity while minimizing potential host toxicity. Finally, clinical trials will be necessary to confirm efficacy, safety, and optimal dosing in pwCF and other non-CF bronchiectasis patients with drug-resistant pulmonary infections.

## 5. Conclusions

OMN51 represents a first-in-class novel approach for combating MDR *P. aeruginosa* infections in pwCF, selectively disrupting the bacterial membrane, resulting in a potent antimicrobial effect. The in vitro findings in this study provide a proof of concept that OMN51 exerts robust antimicrobial activity against both sensitive and resistant strains of *P. aeruginosa* in clinical isolates from sputum cultures of pwCF. OMN51’s in vitro efficacy and resistance-evading mechanism make it a compelling candidate for further development. Further studies are ongoing or planned to address pharmacokinetic and pharmacodynamic properties, in vivo validation, safety, formulation optimization, and clinical trial design to assess the potential of OMN51 as a novel antimicrobial therapy. If successful, OMN51 could significantly expand treatment options for pwCF and non-CF bronchiectasis patients suffering from chronic drug-resistant lung infections, ultimately improving clinical outcomes and quality of life.

## Figures and Tables

**Table 1:** OMN51 MIC data and resistance profiling on 56 clinical isolates of *P. aeruginosa* bacteria.

					Characterization											
No.	Age	Gender	Resistance Pattern	OMN51 MIC [µg/mL]	ATM	FDC	CAZ	CZA	CT	CIP	IPM	MEM	OFL	PIP	TZP	TOB
1	29	M	Susceptible	4	S	NA	S	S	NA	S	S	S	S	S	S	S
2	NA	NA	Susceptible	4	S	NA	S	S	NA	S	S	S	S	S	S	S
3	NA	NA	Susceptible	4	S	NA	S	S	NA	S	S	S	S	S	S	S
4	39	F	Susceptible	8	S	NA	S	S	NA	S	S	S	S	S	S	S
5	52	F	Susceptible	8	S	NA	S	S	NA	S	S	S	S	S	S	S
6	52	F	Susceptible	16	S	NA	S	S	NA	S	S	S	S	S	S	S
7	58	F	Susceptible	8	S	NA	S	S	NA	S	S	S	S	S	S	S
8	39	F	Susceptible	4	S	NA	S	S	NA	S	S	S	S	S	S	S
9	75	M	Susceptible	4	S	NA	S	S	NA	S	S	S	S	S	S	S
10	NA	NA	Susceptible	8	S	NA	S	S	NA	S	S	S	S	S	S	NA
11	70	F	Susceptible	8	S	NA	S	S	NA	S	S	S	S	S	S	NA
12	39	F	ND	4	I	NA	S	S	NA	S	S	S	S	S	S	S
13	51	M	ND	8	S	NA	S	S	S	S	I	S	S	S	S	I
14	25	M	ND	8	S	NA	S	S	NA	S	S	S	I	I	I	S
15	62	F	ND	8	S	NA	S	S	NA	I	S	S	R	S	S	S
16	38	M	ND	4	S	S	S	S	S	S	R	I	I	S	S	S
17	46	F	ND	4	S	NA	S	S	NA	R	S	S	R	S	S	NA
18	34	M	ND	8	S	NA	S	S	NA	R	S	S	R	S	S	NA
19	34	M	ND	16	S	NA	S	S	NA	R	S	S	R	S	S	NA
20	38	M	ND	4	S	NA	S	S	NA	I	R	S	R	S	S	S
21	40	F	ND	4	S	S	S	S	S	R	R	S	R	S	S	S
22	38	M	ND	4	S	S	S	S	S	R	R	S	R	S	S	S
23	36	F	ND	4	S	S	S	S	NA	R	R	S	R	S	S	S
24	40	F	ND	4	S	NA	S	S	NA	R	R	S	R	S	S	S
25	36	F	MDR	8	S	NA	S	S	NA	R	R	S	R	R	S	S
26	22	F	MDR	16	R	NA	S	S	NA	R	S	S	R	S	S	R
27	63	F	MDR	16	R	S	S	S	S	R	S	R	R	S	S	R
28	40	F	MDR	4	S	S	R	S	NA	R	R	R	R	R	S	S
29	31	F	MDR	16	R	S	R	R	NA	R	S	R	R	S	S	R
30	32	F	MDR	4	R	S	R	S	S	R	R	R	R	R	R	S
31	51	M	MDR	16	I	R	R	S	I	R	R	I	R	S	S	R
32	51	M	MDR	8	R	R	R	S	R	R	R	I	R	S	S	R
33	38	M	MDR	8	R	S	R	S	S	R	R	R	R	R	R	R
34	38	M	MDR	4	R	S	R	S	S	R	R	R	R	R	R	R
35	36	F	MDR	16	R	R	R	S	S	R	R	R	R	R	R	R
36	36	F	MDR	8	R	S	R	R	S	R	R	R	R	R	R	R
37	30	M	MDR	16	R	S	R	I	R	R	R	R	R	I	I	R
38	36	F	MDR	16	R	R	R	S	R	R	R	R	R	R	I	R
39	63	F	MDR	16	R	S	R	R	R	R	R	R	R	R	R	R
40	34	M	MDR	8	R	S	R	R	R	R	R	R	R	R	R	R
41	NA	NA	MDR	16	R	S	R	R	R	R	R	R	R	R	R	R
42	48	F	MDR	16	R	S	R	R	R	R	R	R	R	R	R	R
43	32	F	MDR	16	R	S	R	R	R	R	R	R	R	R	R	R
44	32	F	MDR	8	R	I	R	R	NA	R	R	R	R	R	R	R
45	48	F	MDR	16	R	I	R	R	I	R	R	R	R	R	R	R
46	63	F	MDR	4	R	R	R	R	NA	R	R	R	R	R	R	R
47	36	F	MDR	8	R	R	R	R	R	R	R	R	R	R	R	R
48	34	M	MDR	16	R	R	R	R	R	R	R	R	R	R	R	R
49	32	F	MDR	8	R	R	R	R	R	R	R	R	R	R	R	R
50	32	F	MDR	16	R	R	R	R	R	R	R	R	R	R	R	R
51	39	M	MDR	16	R	R	R	R	R	R	R	R	R	R	R	R
52	36	F	MDR	8	R	R	R	R	R	R	R	R	R	R	R	R
53	31	F	MDR	16	R	R	R	R	R	R	R	R	R	R	R	R
54	31	F	MDR	16	R	R	R	R	R	R	R	R	R	R	R	R
55	63	F	MDR	16	R	R	R	R	R	R	R	R	R	R	R	R
56	22	F	MDR	8	R	R	R	R	R	R	R	R	R	R	R	R

Abbreviations: ND: not determined; MDR: multi-drug-resistant; NA: not available; S: susceptible (green shading); I: intermediate (orange shading); R: resistant (red shading); ATM: Aztreonam; FDC: Cefiderocol; CAZ: Ceftazidime; CZA: Ceftazidime–Avibactam; CT: Ceftolozane–Tazobactam; CIP: Ciprofloxacin; IPM: Imipenem; MEM: Meropenem; OFL: Ofloxacin; PIP: Piperacillin; TZP: Piperacillin–Tazobactam; TOB: Tobramycin. Resistance pattern refers to the breakpoints defined by CLSI, which categorize bacteria as susceptible, intermediate, or resistant based on the MIC.

**Table 2:** Summary susceptibility data for all *P. aeruginosa* isolates tested.

Antibiotic	CLSI Breakpoints (S|I|R)	Percentage of Clinical Isolates by Susceptibility Category for Each Antibiotic (%)			
		S	I	R	NA
**OFL**	≤2|4|≥8	23.2	3.6	73.2	0.0
**CIP**	≤0.5|1|≥2	26.8	3.6	69.6	0.0
**IPM**	≤2|4|≥8	35.7	1.8	62.5	0.0
**ATM**	≤8|16|≥32	44.6	3.6	51.8	0.0
**CAZ**	≤8|16|≥32	48.2	0.0	51.8	0.0
**TOB**	≤1|2|≥4	39.3	1.8	51.8	7.1
**MEM**	≤2|4|≥8	44.6	5.4	50.0	0.0
**PIP**	≤16|32|≥64	50.0	3.6	46.4	0.0
**TZP**	≤16/4|32/4|≥64/4	53.6	5.4	41.1	0.0
**CZA**	≤8/4|-|≥16/4	62.5	1.8	35.7	0.0
**CT**	≤4/4|8/4|≥16/4	17.9	3.6	32.1	46.4
**FDC**	≤4|8|≥16	30.4	3.6	26.8	39.3

Antibiotic abbreviations: ATM: Aztreonam; FDC: Cefiderocol; CAZ: Ceftazidime; CZA Ceftazidime–Avibactam; CT: Ceftolozane–Tazobactam; CIP: Ciprofloxacin; IPM: Imipenem; MEM: Meropenem; OFL: Ofloxacin; PIP: Piperacillin; TZP: Piperacillin–Tazobactam; TOB: Tobramycin. Susceptibility abbreviations: S: susceptible; I: indeterminate; R: resistant; NA: not available.

## Data Availability

The datasets presented in this article are not readily available because the datasets are part of an ongoing study of a proprietary compound.

## Abbreviations

The following abbreviations are used in this manuscript:

AMP	antimicrobial peptide
CLSI	Clinical and Laboratory Standards Institute
CFU	colony-forming units
CF	cystic fibrosis
MIC	minimum inhibitory concentration
MDR	multi-drug-resistant
pwCF	people with cystic fibrosis
RBC	red blood cell
